# Neuroprotection and immunomodulation following intraspinal axotomy of motoneurons by treatment with adult mesenchymal stem cells

**DOI:** 10.1186/s12974-018-1268-4

**Published:** 2018-08-14

**Authors:** A. B. Spejo, G. B. Chiarotto, A. D. F. Ferreira, D. A. Gomes, R. S. Ferreira, B. Barraviera, A. L. R. Oliveira

**Affiliations:** 1 Department of Structural and Functional Biology, Institute of Biology, University of Campinas, Campinas, SP Brazil; 2 Department of Biochemistry and Immunology, Institute of Biological Sciences, Federal University of Minas Gerais, Belo Horizonte, MG Brazil; 3 Center for the Study of Venoms and Venomous Animals (CEVAP), São Paulo State University (UNESP), Botucatu, SP Brazil

**Keywords:** Spinal cord, Funiculus cut, Motoneuron, Astrogliosis, Microglial reaction, Immunomodulation

## Abstract

**Background:**

Treatment of spinal cord injury is dependent on neuronal survival, appropriate synaptic circuit preservation, and inflammatory environment management. In this sense, mesenchymal stem cell (MSC) therapy is a promising tool that can reduce glial reaction and provide trophic factors to lesioned neurons.

**Methods:**

Lewis adult female rats were submitted to a unilateral ventral funiculus cut at the spinal levels L4, L5, and L6. The animals were divided into the following groups: IA (intramedullary axotomy), IA + DMEM (Dulbecco’s modified Eagle’s medium), IA + FS (fibrin sealant), IA + MSC (10^6^ cells), and IA + FS + MSC (10^6^ cells). Seven days after injury, qPCR (*n* = 5) was performed to assess gene expression of VEGF, BDNF, iNOS2, arginase-1, TNF-α, IL-1β, IL-6, IL-10, IL-4, IL-13, and TGF-β. The cellular infiltrate at the lesion site was analyzed by hematoxylin-eosin (HE) staining and immunohistochemistry (IH) for Iba1 (microglia and macrophage marker) and arginase-1. Fourteen days after injury, spinal alpha motor neurons (MNs), evidenced by Nissl staining (*n* = 5), were counted. For the analysis of astrogliosis in spinal lamina IX and synaptic detachment around lesioned motor neurons (GAP-43-positive cells), anti-GFAP and anti-synaptophysin immunohistochemistry (*n* = 5) was performed, respectively. Twenty-eight days after IA, the gait of the animals was evaluated by the walking track test (CatWalk; *n* = 7).

**Results:**

The site of injury displayed strong monocyte infiltration, containing arginase-1-expressing macrophages. The FS-treated group showed upregulation of iNOS2, arginase-1, proinflammatory cytokine (TNF-α and IL-1β), and antiinflammatory cytokine (IL-10, IL-4, and IL-13) expression. Thus, FS enhanced early macrophage recruitment and proinflammatory cytokine expression, which accelerated inflammation. Rats treated with MSCs displayed high BDNF-positive immunolabeling, suggesting local delivery of this neurotrophin to lesioned motoneurons. This BDNF expression may have contributed to the increased neuronal survival and synapse preservation and decreased astrogliosis observed 14 days after injury. At 28 days after lesion, gait recovery was significantly improved in MSC-treated animals compared to that in the other groups.

**Conclusions:**

Overall, the present data demonstrate that MSC therapy is neuroprotective and, when associated with a FS, shifts the immune response to a proinflammatory profile.

**Electronic supplementary material:**

The online version of this article (10.1186/s12974-018-1268-4) contains supplementary material, which is available to authorized users.

## Background

Spinal cord injury is an incapacitating condition usually associated with loss of sensory, motor, and autonomic function, greatly reducing quality of life and future prospects [1, 2]. In recent decades, a great amount of effort has been dedicated to better understanding the cellular events following central nervous system (CNS) injury. As of yet, spinal cord lesion treatment in the clinic is insufficient, and complex strategies are clearly necessary to promote functional recovery. In this way, specific animal models of spinal cord injury may provide fundamental information regarding the inflammatory reaction and particular cellular responses that cannot be investigated in extensive lesions.

We have previously shown that ventral root crush results in degeneration of spinal motoneurons, accompanied by astrocyte reactivity and microglial reaction. Such cell loss and inflammation profoundly affects the spinal cord circuitry, decreasing immunoreactivity for presynaptic markers, such as synaptophysin and synapsin [3]. Importantly, mesenchymal stem cell therapy has been shown to improve walking parameters, including strength during the support and propulsion phases of ambulation.

Penetrating spinal cord lesions are associated with persistent breakage of the blood-brain barrier, allowing plasma proteins together with blood-borne cells to gain access to the CNS environment [4, 5]. In this context, the overlap of neural damage and the immune response may provide unexpected positive regenerating responses, as suggested in different instances [4–10].

The experimental model of ventral funiculus cut at the lumbar intumescence allows for the study of motoneuron regeneration after proximal axotomy adjacent to the CNS/PNS interface. In this scenario, the use of immunomodulatory approaches can be tested and properly evaluated in long-term experiments.

Ventral funiculus injuries result in motoneuron axotomy particularly close to the cell body, thereby inducing motoneuron degeneration [11] and synapse loss. Thus, Lindå et al. [12] have demonstrated a transient loss of inputs by alpha-motoneurons following proximal axotomy, peaking at 3 weeks postoperatively, representing only 13% of boutons on the cell soma. Importantly, excitatory inputs are preferentially eliminated, leading to a ratio of one to six terminals in relation to inhibitory boutons. Loss of inhibitory and excitatory synapses can also be depicted following ventral root crush [3]. However, a general immunohistochemistry evaluation at the ventral horn indicated 60% loss of both elements, reinforcing the significant effects of proximal axotomy on spinal cord circuits [3].

Glial reaction and scar formation within the white matter add more complexity to the eventual regeneration of axons towards the ventral roots. Nevertheless, surviving motoneurons have been demonstrated to be able to produce sprouts that, at least in part, overcome the inhibitory barrier imposed by astrogliosis and grow within the PNS to reach the target muscles [13–15]. In this regard, immunomodulation by stem cell therapy seems to positively influence regeneration and motoneuron survival following root avulsion, facilitating growing axons to overcome scar tissue formed after spinal cord lesion [16–19].

In the case of perforating injuries, cavitation occurs, increasing the physical barrier that hampers axon elongation. Thus, the use of bioresorbable frameworks may improve the regenerative process. Herein, we applied a fibrin sealant (FS), a biopolymer scaffold [20], to fill the gap formed by the penetrating lesion and to retain a portion of the applied stem cells.

Overall, the results of the present work demonstrate that mesenchymal stem cell therapy is neuroprotective, leading to increased neuronal survival, decreased astrogliosis, preservation of spinal circuits, and functional recovery.

## Methods

### Experimental design

**Fig. 1:**
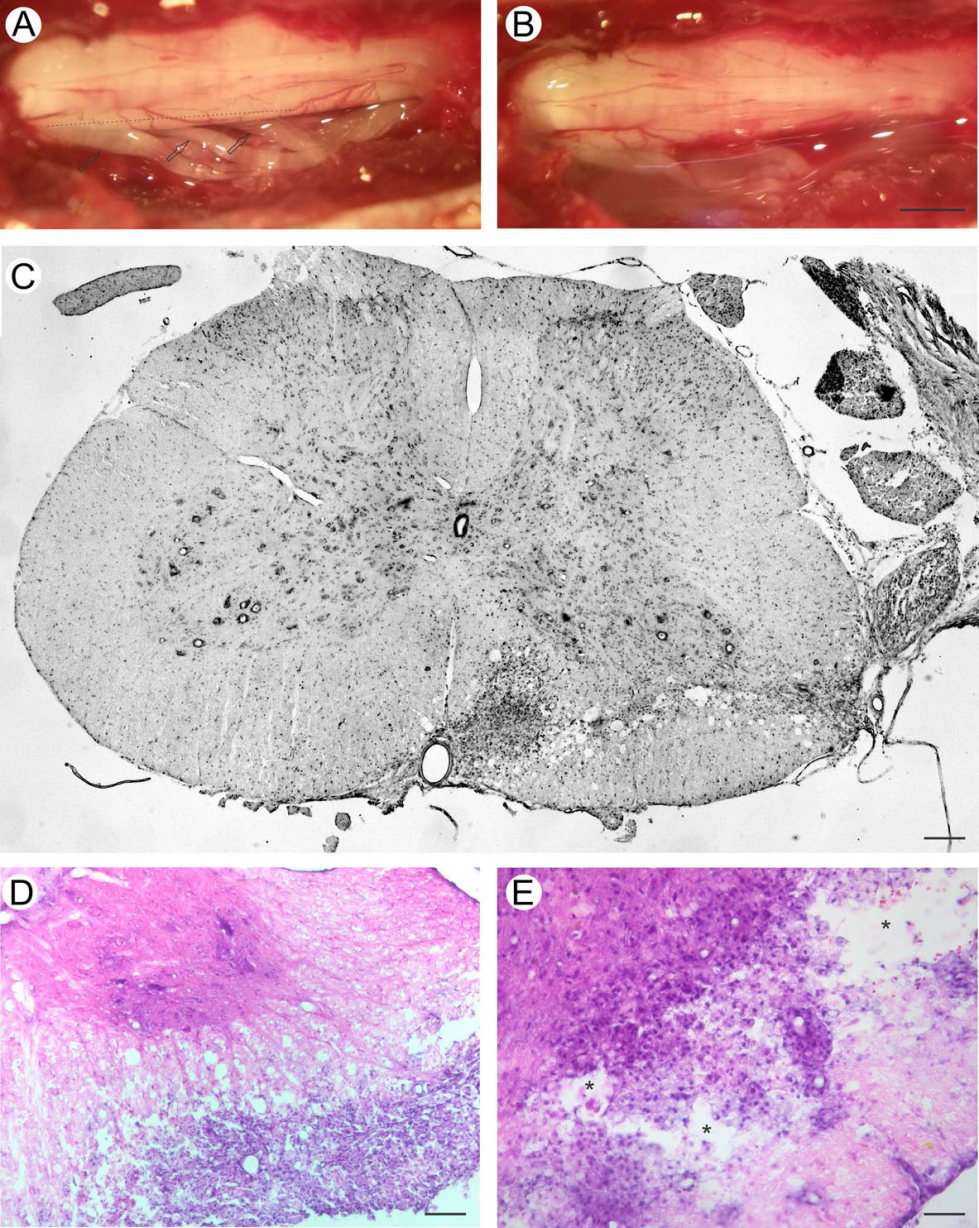
**a** Lesion site evidenced by the presence of L4, L5, and L6 ventral roots (arrows). The longitudinal incision was made above these roots (dotted line). Scale bar = 1.0 mm. **b** Lesion site after MSC application. Note that the region is completely covered by the DMEM + MSC solution. **c** Cresyl violet-stained spinal cord. There is a wide unilateral lesion at the ventral funiculus and fewer motor neurons in the ipsilateral ventral horn than in the contralateral side. Scale bar = 200 μm. **d** HE staining showing the ventral funiculus region. The notorious presence of several nuclei stained with eosin, showing cellular infiltration at the lesion region. Scale bar = 50 μm. **e** High magnification of HE staining. Cavitation points (asterisk) and “Gitter” cells are noted. Scale bar = 100 μm

Lewis adult female rats (~ 200 g, 7–11 weeks old) were submitted to a unilateral ventral funiculus cut, leading to an intraspinal axotomy of motoneurons on the spinal levels L4, L5, and L6. The animals were divided into the following groups: IA (intramedullary axotomy), IA + DMEM (Dulbecco’s modified Eagle’s medium), IA + FS, IA + MSC, and IA + FS + MSC. Lesion completeness was histologically determined based on the location and extension of the ventral funiculus cut in spinal cord sections stained with cresyl violet (Nissl staining). Motoneurons were identified by the location and size at lamina IX of Rexed, similar to as described in the literature [3, 16, 18, 19, 21–23]. Based on the histological evaluation, only animals with complete ventral funiculus cut (reaching the anterior median fissure) and without direct damage to the spinal gray matter were included in the experimental groups. Figure 1c shows the spinal cord histology after a complete unilateral ventral funiculus cut. The contralateral side of the lesion was used as an intern control. For the qPCR experiments, animals without injury were included as a control.

All procedures were carried out with the approval of the Ethics Committee on Animal Experimentation of the University of Campinas, SP, Brazil (CEUA/UNICAMP, protocol 3081-1) and in accordance with the ethical principles regulated by the National Council of Animal Experimentation (CONCEA).

Inflammation and trophic factor production were studied by qPCR (*n* = 5 in each group) 7 days post injury (dpi), and the production of BDNF by the MSCs was examined by immunohistochemistry (IH) at 14 dpi. Neuronal survival after lesion was evaluated by Nissl staining (*n* = 5), and synaptic detachment on the surface of axotomized motoneurons and astrogliosis in lamina IX were assessed by IH (*n* = 5) at 14 dpi. Functional recovery (*n* = 7), using the CatWalk system, was evaluated at 28 dpi.

### Fibrin sealant preparation

The HFS was provided by the Center for the Study of Venoms and Venomous Animals (CEVAP) from São Paulo State University (UNESP), São Paulo, Brazil. Its components and application formula are stated in the patents with record numbers BR1020140114327 and BR1020140114360. The FS polymerizes some seconds after the mixing of its three components: fibrinogen derived from buffalo (*Bubalus bubalis*) blood, 25 mM calcium chloride, and thrombin-like protein obtained from rattlesnake (*Crotalus durissus terrificus*) venom [24–26].

### Mesenchymal stem cell preparation

The MSCs were obtained from the bone marrow of transgenic Lewis rats expressing enhanced green fluorescent protein (EGFP) (LEW-Tg EGFP F455/Rrrc). Homozygous EGFP-positive rats, 4 to 6 weeks old, were euthanized with a lethal dose of halothane. The bone marrow was extracted from the tibiae and femurs and resuspended in DMEM (Dulbecco’s modified Eagle medium—Gibco, Grand Island, NY, USA). The bone marrow cells were plated into 75-cm^2^ flasks containing DMEM supplemented with 10% FBS (fetal bovine serum—Gibco, Grand Island, NY, USA). Cells were cultured until the fourth passage and applied to animals of the experimental groups IA + MSC and IA + FS + MSC.

### MSC phenotyping by flow cytometry

MSCs were characterized by the presence of the following cell surface molecules: CD90, CD54, CD73, and RT1A (MHC I), considered as positive “markers” of these cells [27, 28]. As a negative control, antibodies to CD45 (hematopoietic stem cell marker), CD11b/c (monocyte and macrophage markers), and CD34 (hematopoietic stem cell and endothelial cells marker) were used. After trypsin treatment, approximately 10^6^ cells were incubated for 30 min at 4 °C with the primary antibodies (Additional file 1: Table S1) against CD90, CD54, CD73, RT1A, CD45, CD11b/c, and CD34. Cells were washed with phosphate-buffered saline (PBS) and incubated with Alexa Fluor 488-labeled secondary antibody (Molecular Probes, 1:500) for 30 min at 4 °C. Cells were rewashed in PBS, fixed in 2% formaldehyde, and analyzed on the flow cytometer (Guava® easyCyte ™ 6-2L Flow Cytometer, Millipore, USA). As a negative fluorescence control, the secondary antibody was also added to cells not labeled with the primary antibody. Non-labeled cells were fixed and used to generate the graph of size versus granularity and establish the cell population to be analyzed. The data was analyzed using the FlowJo 7.5.6 software.

### Ventral funiculus cut plus heterologous fibrin sealant and mesenchymal stem cell application

The animals were anesthetized with a combination of xylazine (Anasedan®, 10 mg/kg, Sespo Indústria e Comércio Ltda, Paulinia, SP, Brazil) and ketamine (Dopalen®, 90 mg/kg, Sespo Indústria e Comércio Ltda, Paulinia, SP, Brazil). A dorsal incision parallel to the spine was performed at the thoracic region, and the paravertebral muscles were removed. A hemilaminectomy of two vertebrae was performed, exposing the lumbar intumescence. The dura mater was sectioned longitudinally, and the ventral spinal roots L4, L5, and L6 were identified. Using a microscalpel, a unilateral longitudinal incision at the ventral funiculus was made approximately 0.5 mm dorsal to the ventral surface of the spinal cord and extended approximately 2 mm lateral-medial, almost reaching the anterior median fissure. The DMEM, MSCs, and FS were applied directly to the surface of the injured spinal cord, immediately after the lesion, using a micropipette (Fig. 1a, b). The IA + DMEM group received 6 μl of DMEM. For the IA + FS group, the FS was prepared by mixing its three components on the surface of the spinal cord including 3 μl fibrinogen, 2 μl of 25 mM calcium chloride, and 1 μl of thrombin-like protein. For the IA +MSC group, 10^6^ MSCs diluted in DMEM were applied to the spinal cord in a total volume of 6 μl. For the IA + FS + MSC group, 10^6^ MSCs diluted in DMEM in a total volume of 6 μl were mixed with the fibrinogen component of the FS, and the other components were applied as in the IA + FS group. After the surgical procedures, the muscles and skin were sutured in layers, and the animals were kept in the animal house for 7, 14, or 28 days.

### Specimen preparation

The animals were anesthetized as previously described and submitted to a thoracotomy. The vascular system was transcardially perfused with cold PBS (pH 7.4). To obtain specimens for RT-qPCR, the left, right, ventral, and dorsal spinal regions were separated. The ventral segment on the ipsilateral side to the lesion was placed in a microtube and snap-frozen in liquid nitrogen. The same area of noninjured spinal cords was used as a control. The specimens were stored in a − 80 °C freezer. For histological analysis, after perfusion with PBS, the animals were perfused with 4% formaldehyde in 0.1 M sodium phosphate buffer (PB), pH 7.4. The lesioned region of the spinal cord was dissected, postfixed overnight at 4 °C, and then subjected to gradually increased concentrations of sucrose (10, 20, and 30% for 24 h each). The spinal cords were then embedded into Tissue-Tek O.C.T (Sakura Finetek USA, Inc., Torrance, CA, USA) and frozen at − 35 to − 40 °C. Transverse sections (12 μm thick) were obtained in a cryostat (Micron, HM25, USA) and stored at − 20 °C.

### Hematoxylin-eosin and Nissl staining (neuronal survival)

For a histological characterization of the inured anterior funiculus and analysis of inflammatory cell infiltration, hematoxylin-eosin (HE) staining was performed on frozen sections (7 dpi).

Transverse sections of the injured spinal cord (14 dpi) were submitted to Nissl staining (cresyl violet) for neuronal counting after lesion. Motoneurons were identified by their position in the spinal cord (ventral horn, lamina IX), size (substantially larger than interneurons and glial cells), and presence of Nissl substance arranged in polygonal clumps. The motoneurons present in the ventral horn on the ipsilateral and contralateral sides to the injury were counted in alternate sections of each specimen in the injured area of the lumbar intumescence. Only cells with visible nuclei were counted. To correct double counts of neurons, the Abercrombie and Johnson [29] correction formula was used: *N* = *n* × *t*/(*t* + *d*), where *N* is the corrected number of counted neurons, *n* is the number of counted neurons, *t* is the thickness of the sections, and *d* is the mean diameter of the neuron nucleus [29]. The “*d*” value was calculated based on the average diameter of 15 neuronal nuclei for each experimental group and each spinal side (ipsilateral and contralateral side to lesion).

### Immunohistochemistry

The sections were washed with PB and blocked with 3% BSA (bovine serum albumin) in the same buffer for 1 h. The slides were then incubated in a humidified chamber for 3 h with the primary antibodies diluted in 1% BSA and 0.2% Triton in PB. The primary antibodies (Additional file 2: Table S2) used targeted GFAP, synaptophysin, GAP-43, Iba-1, arginase-1, and BDNF. After the sections were washed in PB wash, the secondary antibody CY3 or CY2 (Jackson Immunoresearch, 1:500) according to the affinity to the primary antibody was diluted in 1% BSA and 0.2% Triton in PB and added to the slices, which were then incubated for 45 min in a humidified chamber at room temperature. The slides were washed in PB and mounted on glycerol/PB (3:1).

Immunolabeled slides were observed under a fluorescence microscope (Leica DM5500B). Representative images of lamina IX both ipsi- and contralateral to the lesion were captured per slide (total of three slides per animal, obtained along the injured segments). The quantification of the integrated pixel density, which represents the intensity of protein immunostaining, was performed using ImageJ software (version 1.33u, National Institutes of Health, USA). For the synaptophysin images, eight equidistant areas (80 μm^2^ each) were measured on the surface of each motor neuron (for three contralateral neurons and three ipsilateral neurons per slide). On the ipsilateral side to the lesion, the synaptic covering was only quantified in neurons positive for GAP-43, indicating that the neuron had been axotomized. For the GFAP images, the integrated density of pixels was measured for the entire image. A percentage ratio between the ipsilateral/contralateral side to the lesion was established. The ratio was calculated for each animal and then for each group ± standard error.

### Real-time PCR (RT-qPCR)

The frozen spinal cords were lysed entirely and homogenized in Tryzol (QIAzol Lysis Reagent, Qiagen, Hilden, Germany), and the total RNA was extracted using the kit RNeasy Lipid Tissue Mini Kit (Qiagen), according to the manufacturer’s instructions.

The RNA was eluted in RNase-free water and stored at − 80 °C until the moment of use. The RNA was quantified and analyzed for the absorbance ratios A260/280 nm and A260/230 nm using a nanophotometer. The RNA integrity was evaluated by 1% agarose gel electrophoresis under denaturing conditions. Synthesis of complementary DNA (cDNA) was performed using the RevertAid H Minus First Strand cDNA Synthesis Kit (Thermo Scientific/Fermentas) following the manufacturer’s instructions. The cDNA was synthesized with 2 μg of total RNA using oligo (dT)_18_ as the primer. The cDNA produced was used as template for real-time PCR reactions.

The reactions, always done in triplicate, were carried out using the cDNAs produced, TaqMan®Gene Expression Master Mix (2✕) (Life Technologies/Thermo Fisher Scientific, Carlsbad, CA, USA), RNase-free water, and TaqMan assays (primer + hydrolysis probes) up to a final volume of 20 μL. The assays (Additional file 3: Table S3) used were for GAPDH, HPRT1, iNOS2, Arg-1, TNF-a, IL-1b, IL-6, IL-10, TGF-b, IL-4, IL-13, VEGF, and BDNF. Samples from all animals were tested with two reference genes: GAPDH and HPRT1. The choice of the reference gene was carefully made based on the unchanged expression under all experimental conditions, with HPRT1 shown to be the most adequate. In all cases, negative controls containing RNase-free water instead of the sample were performed. The reactions were performed following the thermocycling indicated by the Master Mix (95 °C for 10 min and 45 cycles of 95 °C for 15 s and 60 °C for 1 min).

Quantitative PCR was performed in the Mx3005P instrument (Agilent, Santa Clara, CA, USA), and the results were calculated using the MxPro software (Agilent). The relative quantification of the genes of interest was calculated using the ΔΔCt method [30].

### Functional recovery (CatWalk)

For the gait recovery analysis, the walking track test was performed using the CatWalk System (Noldus Inc., Wageningen, The Netherlands). Two evaluations (three to four runs acquired for each) were performed before the injury. After the injury, evaluations were made daily 3 to 14 dpi and then three times a week until up to 28 dpi for each animal. As no morphological or molecular differences were found between the groups AI and AI + DMEM, the AI + DMEM group was used as the only control. Parameters such as the paw area, stand (duration of the support phase), and swing speed (speed exerted by the paw when it is not in contact with the glass plate) were analyzed by the ratio between the ipsi-/contralateral hind paws. An average was calculated for the preoperative value, each day per animal and each day per experimental group ± standard error. The peroneal functional index (PFI) was calculated using the following formula described by Bain et al. [31]: PFI = 174.9(EPL − NPL/NPL) + 80.3(ETS − NTS/NTS) − 13.4 (E = experimental side; N = normal side; PL = print length; TS = toe spread/paw width) [31].

### Statistical analysis

Neuronal survival, immunohistochemistry, and RT-qPCR data were evaluated by one-way ANOVA. CatWalk data were analyzed using the two-way ANOVA method (repeated measures mixed model—ANOVA). To evaluate differences between groups, the Bonferroni post-test was used. For the CatWalk data, after two-way ANOVA, differences between curves/groups were determined by the Mann-Whitney test. The level of significance assumed was equal to *P < 0.05, **P < 0.01, and ***P < 0.001.

## Results

### MSC phenotyping (flow cytometry)

Bone marrow MSCs had a spindle-like shape and were adherent to the plastic surface. Flow cytometry analysis showed that a significant percentage of cells expressed CD90 (95.67%), CD54 (95%), CD73 (95.8%), and RT1A (95%), and a very small number of cells expressed the negative markers CD45 (2.06%), CD11b/c (1.69%), and CD34 (2.02%) (Additional file 4: Figure S1). Thus, the MSCs were considered to meet the criteria used to define this cell type [27, 28], constituting a homogeneous cell population that was clearly distinct from the hematopoietic lineage.

### Spinal cord morphology after IA

The cresyl violet staining revealed a large lesion in the ventral funiculus and fewer neurons in the ipsilateral side of the injury compared to in the contralateral side (Fig. 1c). HE staining revealed a significant infiltration of cells at the lesioned ventral funiculus, cavitation spots, and Gitter cells in all experimental groups (Fig. 1d, e).

### Fate of transplanted MSCs

**Fig. 2:**
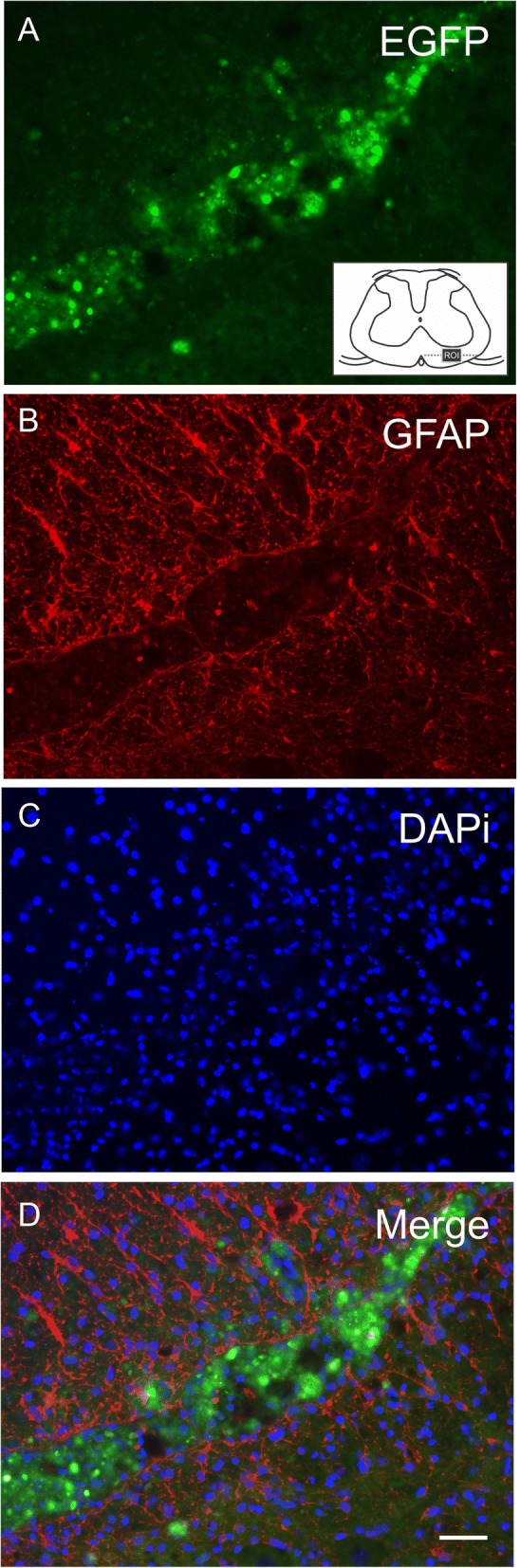
**a**–**d** EGFP-MSCs at the glial scar region in the ventral funiculus at 28 dpi (FS + MSC group image). **a** EGFP-MSCs. Note the region of interest (ROI) in the schematic view of the spinal cord. **b** Anti-GFAP immunostaining. **c** DAPI showing cell nuclei. **d** Co-localization, showing the MSCs at the center of the glial scar delimited by astrocytes. Scale bar = 50 μm

A significant number of EGFP-MSCs were observed up to 28 dpi in the glial scar region of both MSC-treated groups (Fig. 2). Some EGFP-MSCs were also found close to the ventral horn, adjacent to motor neurons.

**Fig. 3:**
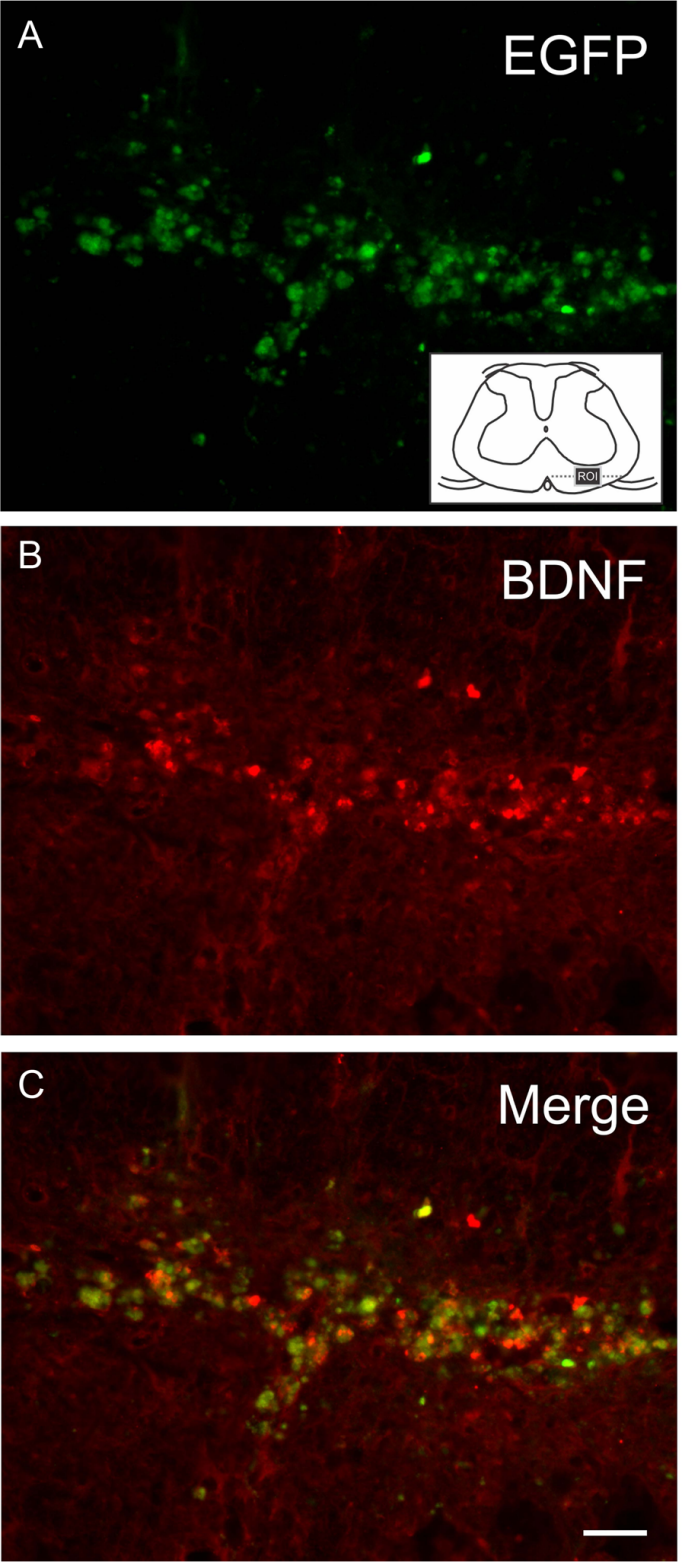
Evaluation of BDNF protein expression by MSCs present in the ventral funiculus at 7 dpi. **a** EGFP-MSCs. Note the region of interest (ROI) in the schematic view of the spinal cord. **b** Anti-BDNF immunostaining. **c** High co-localization between MSC-EGFP and BDNF protein. Scale bar = 100 μm

EGFP-MSCs showed high co-localization with anti-BDNF immunostaining 14 dpi (Fig. 3), revealing that the presence of BDNF was associated with the MSCs.

### Neuronal survival after IA

The absolute number of motor neurons on the contralateral side of the lesion was similar in all experimental conditions (IA 7.84 ± 0.78, IA + DMEM 9.37 ± 1.69, IA + FS 10.20 ± 0.47, IA + MSC 7.94 ± 0.54, IA + FS + MSC 8.94 ± 0.74, mean per section ± standard error). Therefore, the percentage ratio between the number of motor neurons on the ipsilateral and contralateral sides was calculated, reflecting the survival rate of neurons after the lesion.

There was a severe degeneration of injured motor neurons in the IA and IA + DMEM groups at 14 dpi. On the other hand, a higher motor neuronal survival rate was observed in the groups treated with the FS, MSCs, and FS + MSCs.

**Fig. 4:**
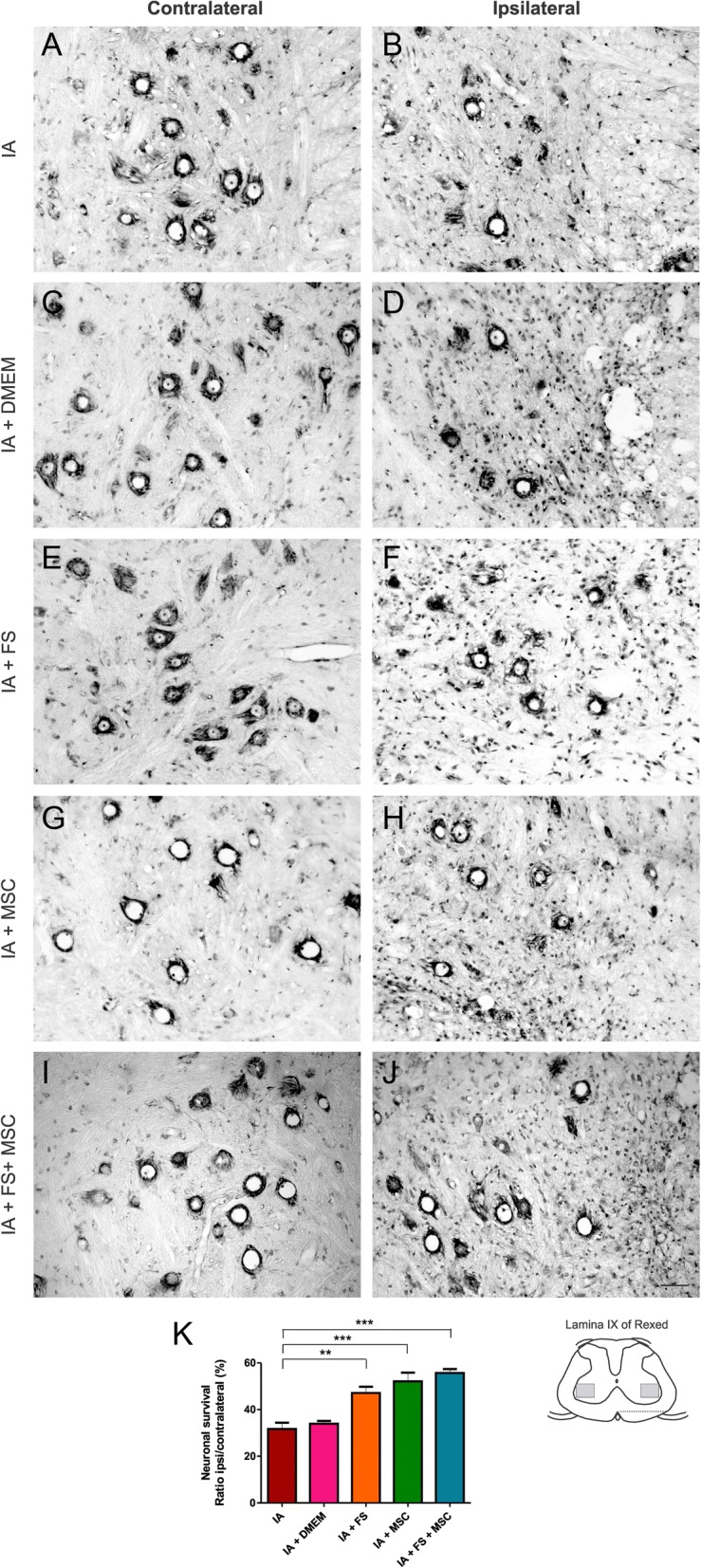
Histological sections of the spinal cord (lamina IX) stained with cresyl violet at 14 dpi. **a**, **c**, **e**, **g**, **i** Contralateral and **b**, **d**, **f**, **h**, **j** ipsilateral sides to the lesion of the groups: **a**, **b** IA; **c**, **d** IA + DMEM; **e**, **f** IA + FS; **g**, **h** IA + MSC; and **i**, **j** IA + FS + MSC. Scale bar = 50 μm. **k** Graphical representation of neuronal survival percentage at 14 dpi

The mean neuronal survival rates ± standard error for each group were as follows: IA 31.67% ± 2.69%, IA + DMEM 33.95% ± 1.20%, IA + FS 47.13% ± 2.63%, IA + MSC 52.10% ± 3.73%, and IA + FS + MSC 55.66% ± 1.73%; *p* < 0.0001 regarding the existence of difference among groups. Significant differences were found between the following groups: IA vs IA + FS, IA + MSC, and IA + FS + MSC; IA + DMEM vs IA + FS, IA + MSC, and IA + FS + MSC. Intergroup significant differences are indicated in the graph of Fig. 4.

### Astrogliosis

At 14 dpi, the contralateral side of the lesion showed low reactivity to GFAP. On the ipsilateral side to IA, there was a considerable increase in GFAP immunolabeling in the ventral horn and ventral funiculus compared to that in the contralateral side.

The MSC-treated animals (IA + MSC and IA + FS + MSC) presented a less intense astroglial reactivity than the animals in the other experimental groups, indicating a possible role of MSCs in the reduction in astrogliosis after injury.

**Fig. 5:**
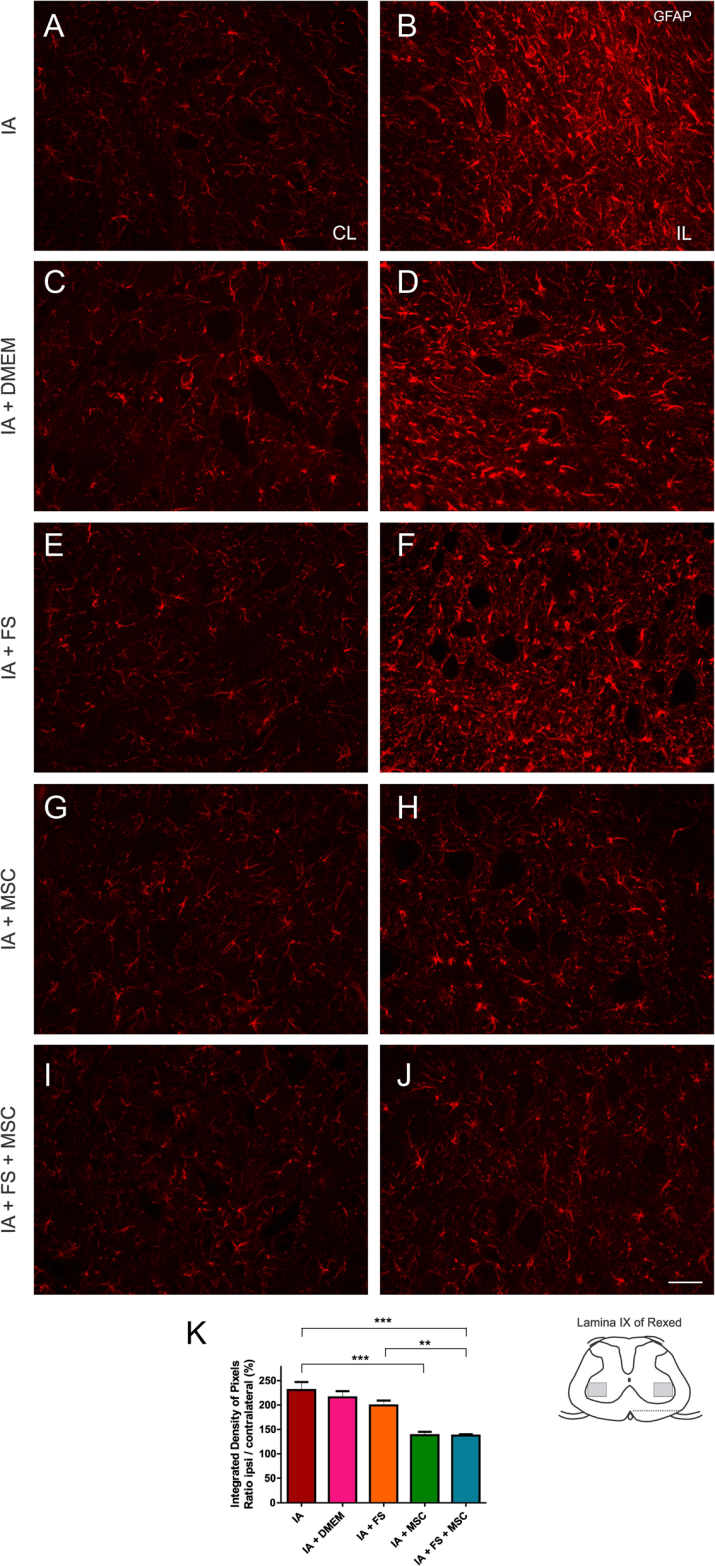
Immunohistochemical analysis of the spinal cord (lamina IX) immunostained for GFAP at 14 dpi. **a**, **c**, **e**, **g**, **i** Contralateral and **b**, **d**, **f**, **h**, **j** ipsilateral sides to the lesion of the groups: **a**, **b** IA; **c**, **d** IA + DMEM; **e**, **f** IA + FS; **g**, **h** IA + MSC, and **i**, **j** IA + FS + MSC. Scale bar = 50 μm. **k** Graphical representation of reactive astrogliosis at 14 dpi

The mean percentage ratio of the integrated density of pixels between the ipsi- and contralateral sides of each group ± standard error were as follows: IA 230.7% ± 16.48%, IA + DMEM 215.7% ± 12.67%, IA + FS 199.1% ± 9.99%, IA + MSC 137.9% ± 7.14%, and IA + FS + MSC 137.4% ± 2.64%; *p* < 0.0001 regarding the existence of difference among groups. Significant differences were found between the following groups: IA vs IA + MSC and IA + FS + MSC; IA + DMEM vs IA + MSC and IA + FS + MSC; IA + FS vs IA + MSC and IA + FS + MSC. Intergroup significant differences are indicated in the graph of Fig. 5.

### Synaptic preservation on the surface of axotomized motor neurons

Intense immunolabeling for synaptophysin was observed adjacent to the cell surface of the motoneurons on the contralateral side of the lesion. In contrast, the intensity of synaptophysin expression was reduced on the surface of axotomized motor neurons, indicating a large synaptic detachment after IA.

VFC promotes the intraspinal axotomy of most motor neurons in the injured spinal segments, but not all motor neurons are affected. In this sense, GAP-43 immunolabeling was required so that only neurons that were axotomized (GAP-43-positive cells) were examined.

Axotomized motoneurons of the MSC-treated groups (IA + MSC and IA + FS + MSC) presented a smaller reduction in terminals in apposition to cell bodies compared to those of the other groups, reflecting a possible effect of the MSCs in the preservation of synapses after lesion.

**Fig. 6:**
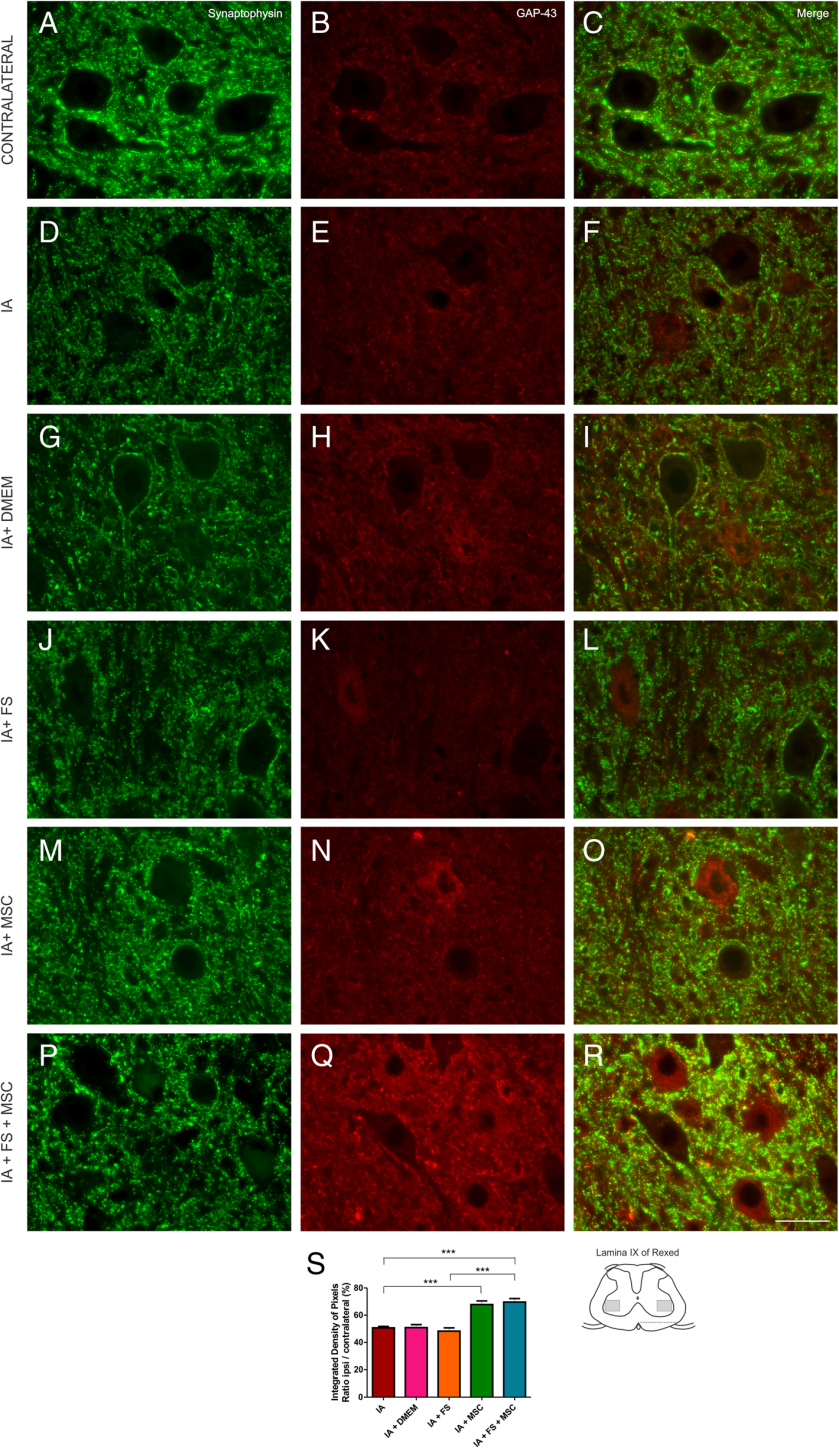
Immunohistochemical analysis of the spinal cord (lamina IX) labeled with anti-synaptophysin and anti-GAP-43 at 14 dpi. **a**–**c** Contralateral and **d**–**r** ipsilateral sides to the lesion of the groups: **d**–**f** IA; **g**–**i** IA + DMEM; **j**–**l** IA + FS; **m**–**o** IA + MSC and **p**–**r** IA + FS + MSC. Scale bar = 50 μm. **s** Graphical representation of synaptic preservation after lesion at 14 dpi

The percentage ratio of the integrated pixel density between the ipsi- and contralateral sides of each group ± standard error was calculated to reflect the synaptic preservation rate after lesion: IA 50.5% ± 1.41%, IA + DMEM 50.87 ± 2.23%, IA + FS 48.32% ± 2.38%, IA + MSC 67.86% ± 2.67%, and IA + FS + MSC 69.60% ± 2.65%; *p* < 0.0001 regarding the existence of difference among groups. Significant differences were found in the following comparisons: IA vs IA + MSC and IA + FS + MSC; IA + DMEM vs IA + MSC and IA + FS + MSC; IA + FS vs IA + MSC and IA + FS + MSC. Intergroup significant differences are indicated in the graph of Fig. 6.

### Acute inflammation after IA

#### Macrophage subtypes

**Fig. 7:**
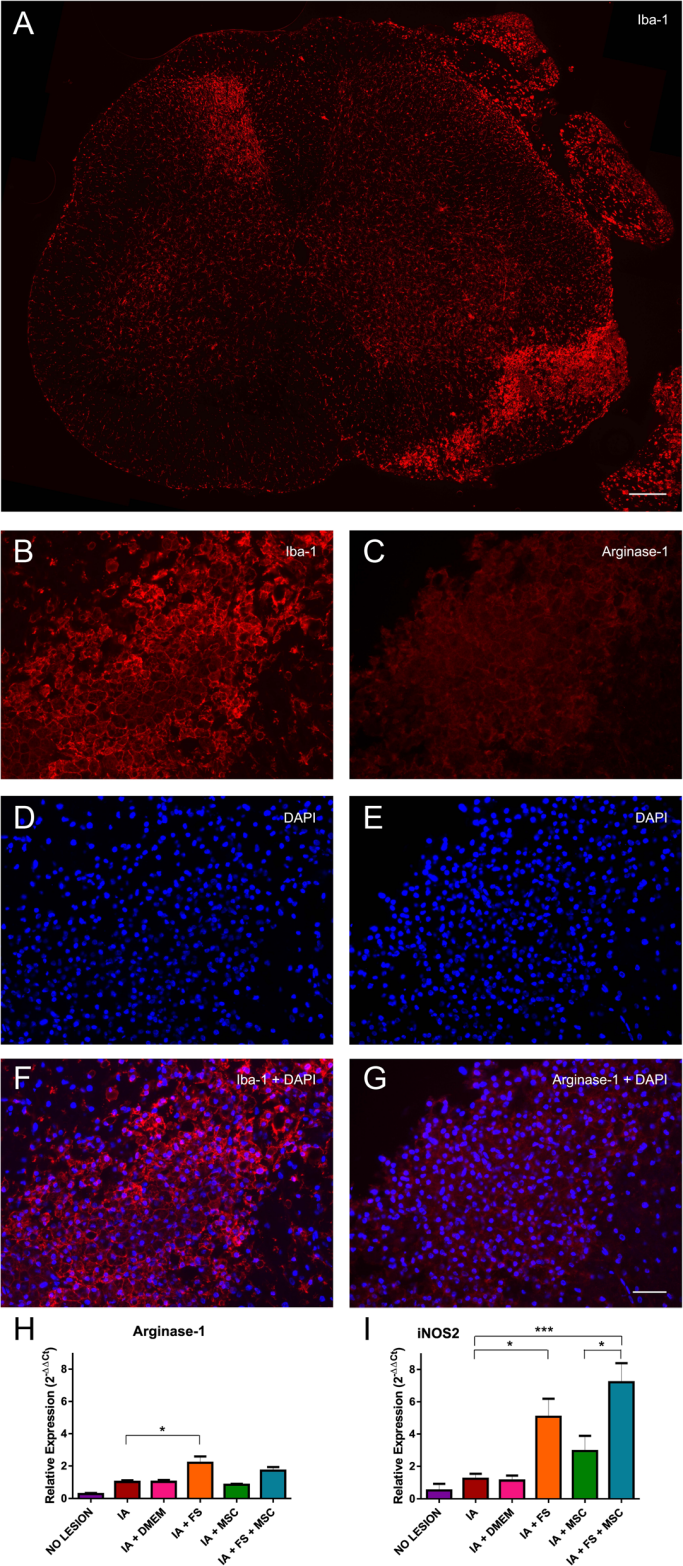
**a** Immunostaining for Iba-1 showing the greater number of macrophages in the lesion region than in the contralateral dorsal region. Scale bar = 200 μm. Lesion site immunostained for **b** Iba-1; **c** arginase-1; **d**, **e**, DAPI to show cell nuclei and **f** overlap of Iba-1 labeling and DAPI and **g** overlap of arginase-1 labeling and DAPI. Scale bar = 50 μm. **h** Relative expression of the gene Arginase-1 at 7 dpi. **i** Relative expression of the gene iNOS2 at 7 dpi

At 7 dpi, a significant portion of the infiltrated cells present at the lesion site was Iba-1 positive and therefore considered macrophages/microglia cells (Fig. 7a). A more intense microglial activity was also observed in the contralateral dorsal horn than in the ipsilateral dorsal horn, probably due to lesion of the spinothalamic afferent pathway located in the ventral funiculus of rats.

Immunostaining revealed that a portion of the macrophages (Iba-1-positive cells) present at the lesion site expressed arginase-1 and, therefore, had an antiinflammatory M2 profile (Fig. 7b–g). Quantification of macrophage subtypes was performed by gene expression analysis of M1 (proinflammatory) and M2 (antiinflammatory) macrophage markers (arginase-1 and iNOS2, respectively) by qPCR at 7 dpi.

Arginase-1 transcript levels were higher in the FS-treated group (without MSC treatment) than in the other experimental groups (no lesion 0.27 ± 0.07, AI 1.02 ± 0.09, AI + DMEM 1.02 ± 0.11, AI + FS 2.19 ± 0.39, AI + MSC 0.85 ± 0.05, AI + FS + MSC 1.70 ± 0.23; *p* < 0.0001 regarding the existence of difference among groups; significant comparisons: no lesion vs IA + FS and IA + FS + MSC; IA vs IA + FS; IA + DMEM vs IA + FS; IA + FS vs IA + MSC). Meaningful intergroup significant differences are indicated in the graph of Fig. 7h.

iNOS2 transcript levels were higher in both groups treated with the FS (with or without MSC treatment) than in the other experimental groups (no lesion 0.52 ± 0.41, AI 1.23 ± 0.31, AI + DMEM 1.13 ± 0.30, AI + FS 5.07 ± 1.12, IA + MSC 2.95 ± 0.94, IA + FS + MSC 7.21 ± 1.18; mean values per group ± standard error; *p* < 0.0001 regarding the existence of difference among groups; significant comparisons: no lesion vs IA + FS and IA + FS + MSC; IA vs IA + FS and IA + FS + MSC; IA + DMEM vs IA + FS and IA + FS + MSC; IA + MSC vs IA + FS + MSC). Meaningful intergroup significant differences are indicated in the graph of Fig. 7i.

#### Proinflammatory cytokines

**Fig. 8:**
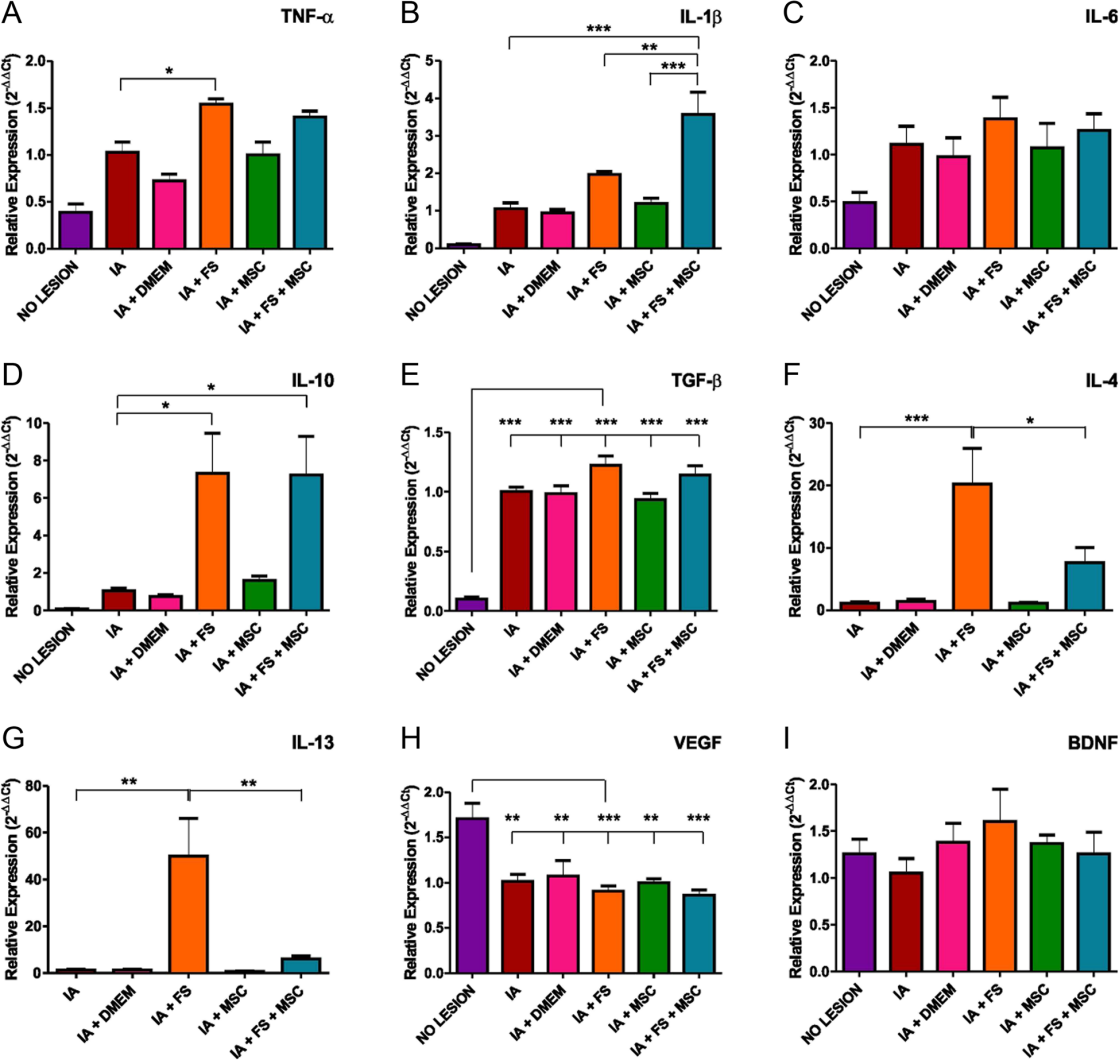
Relative expression of the genes **a** TNF-α, **b** IL-1β, **c** IL-6, **d** IL-10, **e** TGF-β, **f** IL-4, **g** IL-13, **h** VEGF, and **i** BDNF at 7 dpi

TNF-α gene expression was higher in all lesioned groups than in the non-lesioned group. Among the injured groups of animals, the FS-treated groups (with or without MSC therapy) had higher TNF-α expression than those not treated with the FS (no lesion 0.39 ± 0.09, AI 1.03 ± 0.11, AI + DMEM 0.72 ± 0.07, AI + FS 1.54± 0.06, AI + MSC 1.00± 0.14, IA + FS + MSC 1.41 ± 0.06; *p* < 0.0001 regarding the existence of difference among groups; significant comparisons: no lesion vs IA, IA + FS, IA + MSC, and IA + FS + MSC; IA vs IA + FS; IA + DMEM vs IA + FS and IA + FS + MSC; IA + FS vs IA + MSC). Meaningful intergroup significant differences are indicated in the graph of Fig. 8a.

IL-1β gene expression was higher in the FS-treated group than in the other groups and even higher in the IA + FS + MSC group (no lesion 0.10 ± 0.02, AI 1.05 ± 0.16, AI + DMEM 0.94 ± 0.09, AI + FS 1.97 ± 0.08, AI + MSC 1.19 ± 0.14, IA + FS + MSC 3.57 ± 0.59; *p* < 0.0001 regarding the existence of difference among groups; significant comparisons: no lesion vs IA + FS and IA + FS + MSC; IA vs IA + FS + MSC; IA + DMEM vs IA + FS + MSC; IA + FS vs IA + FS + MSC; IA + MSC vs IA + FS + MSC). Meaningful intergroup significant differences are indicated in the graph of Fig. 8b.

The transcript levels for IL-6 were similar among the groups (no lesion 0.49 ± 0.11, AI 1.11 ± 0.20, AI + DMEM 0.98 ± 0.20, AI + FS 1.38 ± 0.23, AI + MSC 1.07 ± 0.26, AI + FS + MSC 1.26 ± 0.18; *p* = 0.0733; Fig. 8c).

#### Antiinflammatory cytokines

The IL-10 transcript levels were higher in the FS-treated groups (with and without MSC therapy) than in the other groups (no lesion 0.08 ± 0.04, AI 1.05 ± 0.15, AI + DMEM 0.75 ± 0.09, AI + FS 7.32 ± 2.15, AI + MSC 1.60 ± 0.25, AI + FS + MSC 7.23 ± 2.07; *p* = 0.0002 regarding the existence of difference among groups; significant comparisons: no lesion vs IA + FS and IA + FS + MSC; IA vs IA + FS and IA + FS + MSC; IA + DMEM vs IA + FS and IA + FS + MSC; IA + FS vs IA + MSC). Meaningful intergroup significant differences are indicated in the graph of Fig. 8d.

Transcripts for TGF-β, a marker of M2C macrophages related to inflammation resolution, were more highly expressed in all experimental groups than in the group without the lesion (no lesion 0.10 ± 0.02, AI 1.00 ± 0.04, AI + DMEM 0.98 ± 0.07, AI + FS 1.22 ± 0.08, AI + MSC 0.93 ± 0.05, IA + FS + MSC 1.14 ± 0.08; *p* < 0.0001 regarding the existence of difference among groups; significant comparisons: no lesion vs IA, IA + DMEM, IA + FS, IA + MSC, and IA + FS + MSC; IA + FS vs IA + MSC). Meaningful intergroup significant differences are indicated in the graph of Fig. 8e.

IL-4 transcript expression was higher in the FS-treated group (without MSC therapy) than in the other groups. The animals given the combined treatment (FS + MSC) did not present the same increased expression, and the animals without lesion did not express this gene (no lesion no expression, AI 1.12 ± 0.28, AI + DMEM 1.41 ± 0.40, IA + FS 20.19 ± 5.73, IA + MSC 1.12 ± 0.13, IA + FS + MSC 7.64 ± 2.43; *p* = 0.0002 regarding the existence of difference among groups; significant comparisons: IA vs IA + FS; IA + DMEM vs IA + FS; IA + FS vs IA + MSC and IA + FS + MSC). Meaningful intergroup significant differences are indicated in the graph of Fig. 8f.

The gene IL-13 was more highly expressed in the FS-treated group than in the other groups. The combined treatment (FS + MSC) did not cause the same increase in expression. The animals without lesion did not express this gene (no lesion no expression, AI 1.27 ± 0.41, AI + DMEM 1.29 ± 0.37, AI + FS 49.94 ± 16.03, AI + MSC 0.69 ± 0.24, IA + FS + MSC 6.02 ± 1.27; *p* = 0.0003 regarding the existence of difference among groups; significant comparisons: IA vs IA + FS; IA + DMEM vs IA + FS; IA + FS vs IA + MSC and IA + FS + MSC). Meaningful intergroup significant differences are indicated in the graph of Fig. 8g.

### Trophic factors

The gene expression of the trophic factors VEGF and BDNF was assessed at 7 dpi. VEGF transcript expression was lower in all lesioned groups than in the uninjured group (no lesion 1.71 ± 0.17, IA 1.01 ± 0.08, AI + DMEM 1.07 ± 0.17, AI + FS 0.91 ± 0.06, AI + MSC 1.00 ± 0.04, AI + FS + MSC 0.86 ± 0.06; *p* = 0.0002 regarding the existence of difference among groups; significant comparisons: no lesion vs IA, IA + DMEM, IA + FS, IA + MSC, and IA + FS + MSC). Meaningful intergroup significant differences are indicated in the graph of Fig. 8h.

BDNF gene expression was similar among all experimental groups (no lesion 1.26 ± 0.16, IA 1.05 ± 0.16, IA + DMEM 1.38 ± 0.20, IA + FS 1.60 ± 0.35, AI + MSC 1.37 ± 0.09, IA + FS + MSC 1.26 ± 0.23; *p* = 0.613; Fig. 8i).

### Functional recovery (Fig. 9)

**Fig. 9:**
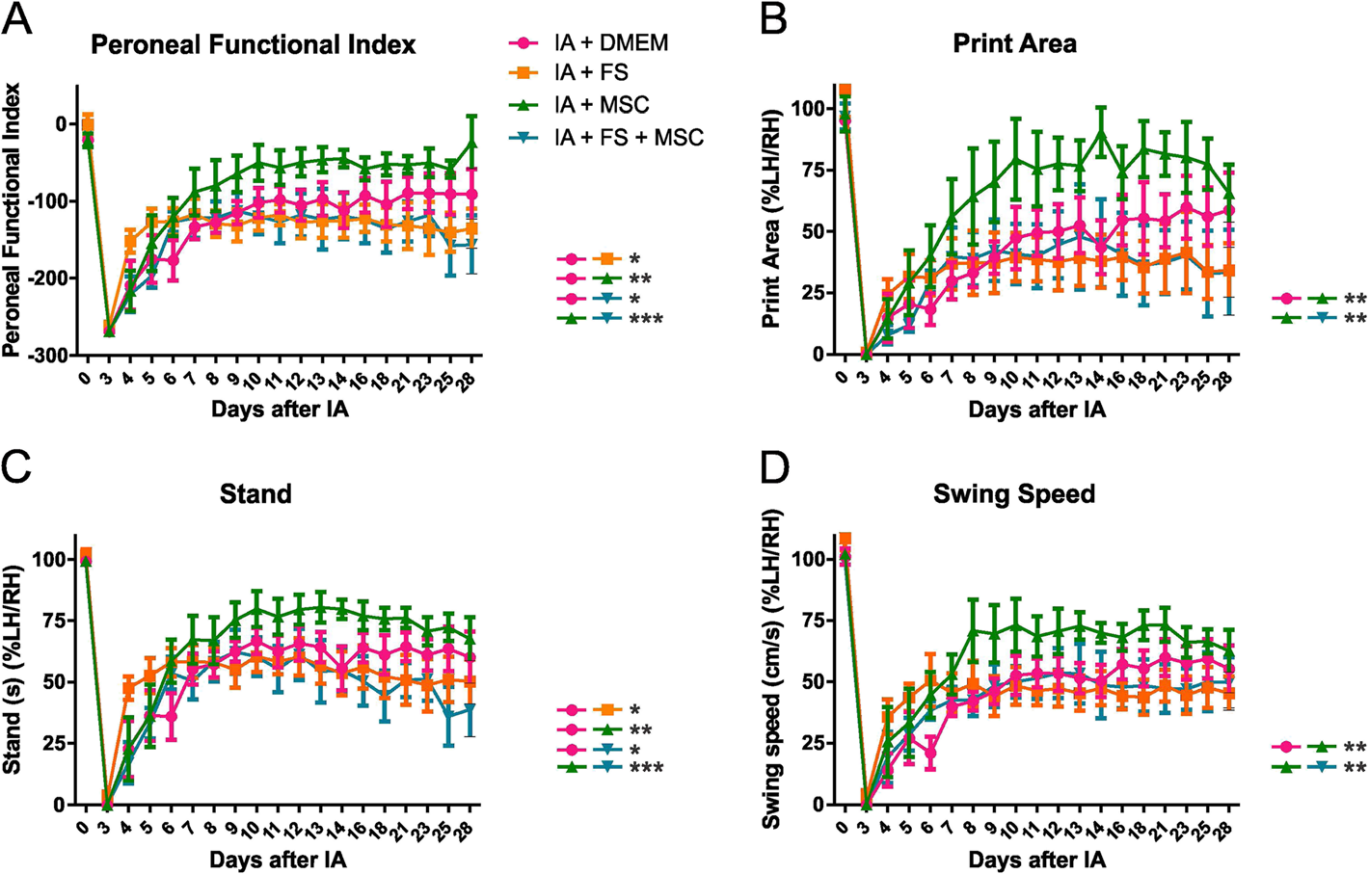
Motor function recovery (CatWalk) at 28 days after injury. **a** Peroneal functional index. **b** Print area ratio between the left and right hind paws. **c** Stand duration ratio between the left and right hind paws. **d** Swing speed ratio between left and right hind paws

CatWalk analysis revealed a significant difference among the experimental groups in the peroneal functional index (*p* < 0.0001 regarding the existence of difference among groups; significant comparisons: IA +DMEM vs IA + FS, IA + MSC, and IA + FS + MSC; AI + FS vs IA + MSC; IA + MSC vs IA + FS + MSC; meaningful intergroup significant differences are indicated in the graph of Fig. 9a), print area (*p* = 0.0128 regarding the existence of difference among groups; statistically different groups: IA + DMEM vs IA + MSC; AI + FS vs IA + MSC; IA + MSC vs IA+ FS + MSC; meaningful intergroup significant differences are indicated in the graph of Fig. 9b), stand duration (duration of the support phase in seconds; *p* = 0.0011 regarding the existence of difference among groups; significant comparisons: IA + DMEM vs IA + FS, IA + MSC, and IA + FS + MSC; AI + FS vs IA + MSC; IA + MSC vs IA + FS + MSC; meaningful intergroup significant differences are indicated in the graph of Fig. 9c), and swing speed (velocity of the paw during the swing phase; *p* = 0.0109 regarding the existence of difference among groups; significant comparisons: IA + DMEM vs IA + MSC; AI + FS vs IA + MSC; IA + MSC vs IA + FS + MSC; meaningful intergroup significant differences are indicated in the graph of Fig. 9d).

For all these parameters, the animals given MSC therapy performed better than those in the other experimental groups, and the FS-treated groups (with or without MSC) performed slightly worse than the control group.

## Discussion

Ventral funiculus cut (VFC) results in the loss of axotomized motoneurons, combined with disorganization of spinal circuits, affecting mostly excitatory synapses [11]. Thus, the loss of inputs combined with the close-to-cell-body section of the motor axon triggers degenerative events. Herein, VFC caused death of 67% of the motoneurons, which is in line with previous works [4, 6, 7, 32]. After ventral root avulsion, in which the motor axons are pulled from the surface of the spinal cord, approximately 80% of motor neurons die within 2 weeks after lesion [33]. The lower percentage of death in the IA model is probably because VFC spares more medially located motoneurons [14] and the axonal traction associated with avulsion causes greater degeneration.

Another parameter that certainly impacts cell survival and synapse stability is the inflammatory process that is closely connected to astroglial and microglial reaction. The present data shows that VFC induces significant astrogliosis and migroglial reactivity, which are observed during the acute phase post injury, i.e., 2 weeks after VFC. In this sense, the glial reaction has been connected to the retraction of inputs to motoneurons following axotomy [34–38]. In previous works, we obtained evidence that by reducing glial reaction, spinal cord circuitry stability could be improved, leading to better functional recovery [16, 17, 22, 23].

Lesion to motoneurons can be promptly demonstrated via a walking track test, which indicates complete loss of motor function soon after injury. The CatWalk system was used herein to assess different gait parameters, as well as the peroneal nerve function index. Overall, a spontaneous partial recovery could be identified over the weeks after lesion, although lesioned rats never fully recovered. Of note, the MSC-treated group achieved the best functional recovery. This may be the combination of different factors, including neuronal survival, decreased glial reaction, local neurotrophic factor release, and improved synaptic stability.

Based on the abovementioned results and in order to overcome the long-term sequelae of spinal cord injury, neuroprotective and immunomodulatory approaches are necessary. The first step towards successful recovery is the enhancement of neuronal survival at the acute phase post lesion. Enhanced neuronal survival has primarily been achieved via treatment with neurotrophic factors [21, 39]. However, robust and continuous delivery of such substances is not cost-effective, produces severe side effects and, most importantly, usually fails to provide guided regrowth of axons towards the nerve roots [40].

Stem cell research has been introduced as a promising tool to overcome most of the unwanted effects of direct neurotrophin therapy. Adult mesenchymal stem cells, for example, produce BDNF and GDNF in sufficient amounts to sustain axotomized motoneurons following ventral root avulsion during the acute phase post injury [16, 23, 41]. Importantly, stem cells migrate towards the injury epicenter, creating a gradient of secreted molecules that guide re-growing axons through the glial scar to the nerve rootlets. The neuroprotection observed after MSC treatment may also be related to a range of other factors that these cells have been shown to produce, including NGF, IGF, GM-CSF, FGF2, TGF, FGF2, HGF, angiopoietin, and VEGF [42–44]. Herein, MSC and FS treatment did not change overall VEGF or BDNF gene expression at 7 days after lesion, although the MSCs displayed high BDNF-positive immunolabeling at 14 days after lesion, suggesting local delivery of neurotrophins to lesioned motoneurons. The presence of the protein not coupled with an enhancement in BDNF gene expression may suggest that increased gene expression may have occurred at an earlier time point than that studied herein.

In a previous work, we introduced the use of a heterologous fibrin sealant that allowed avulsed root reimplantation, functioning as a scaffold for the stem cells and growing axons [23]. Functional results were promising, and no apparent side effects were depicted. Spinal cord-perforating injuries result in tissue loss and cavitation, what may in turn enhance scar tissue deposition. The use of fibrin sealant may help bridge the gap of CNS soft tissue, contributing to greater neuronal survival. The FS-treated group presented increased gene expression of M1 and M2 macrophage markers and antiinflammatory and proinflammatory cytokines, as seen by qRT-PCR. Considering that the fibrin sealant has xenograft fibrinogen in its composition, possibly causing stimulation of the immune response, the fibrin sealant may have generated reactivity of the resident microglia, as well as stimulated recruitment of macrophages derived from monocytes. In this sense, the fibrin sealant could be immunogenic, causing inflammation through proinflammatory mechanisms but also its resolution by antiinflammatory mechanisms. Nevertheless, the fibrin sealant may stimulate macrophage migration and tissue clearance.

Increased influx of macrophages after commercial FS treatment was also reported in comparison to that in untreated animals after T9 hemisection. Macrophage recruitment was higher 3 and 7 days after injury and returned to normal at 28 days post injury [45], in line with the increased recruitment of macrophages seen in the present study 7 days after IA. Moreover, the decrease in the recruitment of macrophages in a later period could be explained by the degradation of the FS and, therefore, an interruption of its action. In the present study, increased expression of pro- and antiinflammatory factors in the FS-treated group did not influence the behavior at the same time point but may have provided a more neuroprotective situation, leading to the greater neuronal survival observed in this group at 14 days after lesion. However, the neuroprotection at this time point was not associated with better motor function, possibly because the sealant is rapidly degraded, what would change the inflammatory profile, but also because neuronal survival alone may not be enough to improve functional recovery. When increased neuronal survival was coupled with synaptic preservation and decreased astrogliosis, as seen in the MSC-treated group, functional recovery was observed.

Animals treated with the FS and MSCs presented a different gene expression pattern than the animals only treated with the FS. The combined treatment caused greater expression of the proinflammatory cytokine IL-1β and lower expression of the antiinflammatory cytokines IL-4 and IL-13 than FS treatment alone. When compared to the control group, the FS + MSC group showed an increase in the expression of proinflammatory M1 macrophages, but unlike the group that received FS alone, there was no increase in the expression of antiinflammatory M2 macrophages. The local environment provided by FS may have altered the properties of the MSCs, generating a predominantly proinflammatory milieu in the combined treatment group, culminating in the absence of motor improvement that was seen in the group treated with MSCs only. Therefore, MSC therapy provided the best regenerative outcome, even in the absence of the fibrin sealant scaffold, through increased neuronal survival, decreased astrogliosis, and synaptic preservation.

## Conclusions

Overall, the present data demonstrate that MSC therapy is neuroprotective and that its association with FS provides an adequate scaffold to retain engrafted cells within the site of the lesion. Nevertheless, such a combined treatment shifted the inflammation pattern to a Th1 profile, reducing sensorimotor performance. Thus, animals treated with MSCs alone showed the best functional performance. In turn, our data reinforce that although the use of combinatorial approaches to treat spinal cord injury is the most desirable, it may provide unexpected results. Future experiments, including clinical trials, will be necessary to further understand and improve recovery from such complex injuries.

## Additional files

Additional file 1: **Table S1.** Primary antibodies used for Flow Cytometry. (DOCX 13 kb)

Additional file 2: **Table S2.** Primary antibodies used for Immunohistochemistry. (DOCX 13 kb)

Additional file 3: **Table S3.** Assays used for qPCR. (DOCX 13 kb)

Additional file 4: **Figure S1.** Phenotypic analysis of the MSCs by flow cytometry. (A-G) Histograms of the mean fluorescence intensity (GRN-HLog: green fluorescence) versus the number of events (counts). (A) CD90. (B) CD54. (C) CD73. (D) MHCl (RT1A). (E) CD45. (F) CD11b/c. (G) CD34. (H) Graphic representation of the percentage of fluorescent cells labeled with each antibody. (PDF 826 kb)

## Abbreviations

BDNF: Brain-derived neurotrophic factor
BSA: Bovine serum albumin
CNS: Central nervous system
CY: Cyanine
DMEM: Dulbecco’s modified Eagle’s medium
EGFP: Enhanced green fluorescent protein
FS: Heterologous fibrin sealant
GAP-43: Growth-associated protein, 43 KDa
GFAP: Glial fibrillary acidic protein
HE: Hematoxylin-eosin
IH: Immunohistochemistry
IL: Interleukin
iNOS: Inducible nitric oxide synthase
MSC: Mesenchymal stem cell
PB: Phosphate buffer
PNS: Peripheral nervous system
TNF-alpha: Tumor necrosis factor alpha
VEGF: Vascular endothelial growth factor
VFC: Ventral funiculus cut
VGLUT: Vesicular glutamate transporter

## References

[1] Hartkopp A, Brønnum-Hansen H, Seidenschnur AM, Biering-Sørensen F (1997). Survival and cause of death after traumatic spinal cord injury. A long-term epidemiological survey from Denmark. Spinal Cord.

[2] Rahimi-Movaghar V, Sayyah MK, Akbari H, Khorramirouz R, Rasouli MR, Moradi-Lakeh M, Shokraneh F, Vaccaro AR (2013). Epidemiology of traumatic spinal cord injury in developing countries: a systematic review. Neuroepidemiology.

[3] Spejo AB, Carvalho JL, Goes AM, Oliveira AL (2013). Neuroprotective effects of mesenchymal stem cells on spinal motoneurons following ventral root axotomy: synapse stability and axonal regeneration. Neuroscience.

[4] Risling M, Linda H, Cullheim S, Franson P (1989). A persistent defect in the blood-brain barrier after ventral funiculus lesion in adult cats: implications for CNS regeneration?. Brain Res.

[5] Risling M, Fried K, Linda H, Cullheim S, Meier M (1992). Changes in nerve growth factor receptor-like immunoreactivity in the spinal cord after ventral funiculus lesion in adult cats. J Neurocytol.

[6] Cullheim S, Wallquist W, Hammarberg H, Linda H, Piehl F, Carlstedt T, Risling M (2002). Properties of motoneurons underlying their regenerative capacity after axon lesions in the ventral funiculus or at the surface of the spinal cord. Brain Res Brain Res Rev.

[7] Skold MK, Marti HH, Lindholm T, Linda H, Hammarberg H, Risling M, Cullheim S (2004). Induction of HIF1alpha but not HIF2alpha in motoneurons after ventral funiculus axotomy-implication in neuronal survival strategies. Exp Neurol.

[8] Gaudet AD, Fonken LK. Glial cells shape pathology and repair after spinal cord injury. Neurotherapeutics. 2018. 10.1007/s13311-018-0630-7. 10.1007/s13311-018-0630-7 PMC6095774 29728852

[9] Gaudet AD, Popovich PG (2014). Extracellular matrix regulation of inflammation in the healthy and injured spinal cord. Exp Neurol.

[10] Schwab JM, Zhang Y, Kopp MA, Brommer B, Popovich PG (2014). The paradox of chronic neuroinflammation, systemic immune suppression, autoimmunity after traumatic chronic spinal cord injury. Exp Neurol.

[11] Linda H, Shupliakov O, Ornung G, Ottersen OP, Storm-Mathisen J, Risling M, Cullheim S (2000). Ultrastructural evidence for a preferential elimination of glutamate-immunoreactive synaptic terminals from spinal motoneurons after intramedullary axotomy. J Comp Neurol.

[12] Hammarberg H, Lidman O, Lundberg C, Eltayeb SY, Gielen AW, Muhallab S, Svenningsson A, Linda H, van Der Meide PH, Cullheim S (2000). Neuroprotection by encephalomyelitis: rescue of mechanically injured neurons and neurotrophin production by CNS-infiltrating T and natural killer cells. J Neurosci.

[13] Risling M, Ochsman T, Carlstedt T, Linda H, Plantman S, Rostami E, Angeria M, Skold MK (2011). On acute gene expression changes after ventral root replantation. Front Neurol.

[14] Risling M, Cullheim S, Hildebrand C (1983). Reinnervation of the ventral root L7 from ventral horn neurons following intramedullary axotomy in adult cats. Brain Res.

[15] Carlstedt T, Cullheim S, Risling M, Ulfhake B: Nerve fibre regeneration across the PNS-CNS interface at the root-spinal cord junction. Brain Res Bull. 1989;22:93–102. 10.1016/0361-9230(89)90133-0 2713720

[16] Barbizan R, Castro MV, Barraviera B, Ferreira RS, Oliveira AL (2014). Influence of delivery method on neuroprotection by bone marrow mononuclear cell therapy following ventral root reimplantation with fibrin sealant. PLoS One.

[17] Barbizan R, Oliveira AL (2010). Impact of acute inflammation on spinal motoneuron synaptic plasticity following ventral root avulsion. J Neuroinflammation.

[18] Vidigal de Castro M, Barbizan R, Seabra Ferreira R, Barraviera B, Leite Rodrigues de Oliveira A (2016). Direct spinal ventral root repair following avulsion: effectiveness of a new heterologous fibrin sealant on motoneuron survival and regeneration. Neural Plast.

[19] Ribeiro TB, Duarte AS, Longhini AL, Pradella F, Farias AS, Luzo AC, Oliveira AL, Olalla Saad ST (2015). Neuroprotection and immunomodulation by xenografted human mesenchymal stem cells following spinal cord ventral root avulsion. Sci Rep.

[20] Biscola NP, Cartarozzi LP, Ulian-Benitez S, Barbizan R, Castro MV, Spejo AB, Ferreira RS, Barraviera B, Oliveira ALR (2017). Multiple uses of fibrin sealant for nervous system treatment following injury and disease. J Venom Anim Toxins Incl Trop Dis.

[21] Blits B, Carlstedt TP, Ruitenberg MJ, de Winter F, Hermens WT, Dijkhuizen PA, Claasens JW, Eggers R, van der Sluis R, Tenenbaum L (2004). Rescue and sprouting of motoneurons following ventral root avulsion and reimplantation combined with intraspinal adeno-associated viral vector-mediated expression of glial cell line-derived neurotrophic factor or brain-derived neurotrophic factor. Exp Neurol.

[22] Araujo MR, Kyrylenko S, Spejo AB, Castro MV, Ferreira Junior RS, Barraviera B, Oliveira ALR (2017). Transgenic human embryonic stem cells overexpressing FGF2 stimulate neuroprotection following spinal cord ventral root avulsion. Exp Neurol.

[23] Barbizan R, Castro MV, Rodrigues AC, Barraviera B, Ferreira RS, Oliveira AL (2013). Motor recovery and synaptic preservation after ventral root avulsion and repair with a fibrin sealant derived from snake venom. PLoS One.

[24] Ferreira Junior RS (2014). Autologous or heterologous fibrin sealant scaffold: which is the better choice?. J Venom Anim Toxins Incl Trop Dis.

[25] Ferreira Junior RS, de Barros LC, Abbade LPF, Barraviera SRCS, Silvares MRC, de Pontes LG, Dos Santos LD, Barraviera B (2017). Heterologous fibrin sealant derived from snake venom: from bench to bedside - an overview. J Venom Anim Toxins Incl Trop Dis.

[26] Barros LC, Soares AM, Costa FL, Rodrigues VM, Fuly AL, Giglio JR, Gallacci M, Thomazini-Santos IA, Barraviera SRCS, Barraviera B, Ferreira Junior RS (2011). Biochemical and biological evaluation of gyroxin isolated from Crotalus durissus terrificus venom. J Venom Anim Toxins Incl Trop Dis.

[27] Delorme B, Ringe J, Gallay N, Le Vern Y, Kerboeuf D, Jorgensen C, Rosset P, Sensebe L, Layrolle P, Haupl T, Charbord P (2008). Specific plasma membrane protein phenotype of culture-amplified and native human bone marrow mesenchymal stem cells. Blood.

[28] Dominici M, Le Blanc K, Mueller I, Slaper-Cortenbach I, Marini F, Krause D, Deans R, Keating A, Prockop D, Horwitz E (2006). Minimal criteria for defining multipotent mesenchymal stromal cells. The International Society for Cellular Therapy position statement. Cytotherapy.

[29] Abercrombie M, Johnson ML (1946). Quantitative histology of Wallerian degeneration: I. Nuclear population in rabbit sciatic nerve. J Anat.

[30] Livak KJ, Schmittgen TD (2001). Analysis of relative gene expression data using real-time quantitative PCR and the 2(−Delta Delta C(T)) method. Methods.

[31] Bain JR, Mackinnon SE, Hunter DA (1989). Functional evaluation of complete sciatic, peroneal, and posterior tibial nerve lesions in the rat. Plast Reconstr Surg.

[32] Lindholm T, Cullheim S, Deckner M, Carlstedt T, Risling M (2002). Expression of neuregulin and ErbB3 and ErbB4 after a traumatic lesion in the ventral funiculus of the spinal cord and in the intact primary olfactory system. Exp Brain Res.

[33] Koliatsos VE, Price WL, Pardo CA, Price DL (1994). Ventral root avulsion: an experimental model of death of adult motor neurons. J Comp Neurol.

[34] Blinzinger K, Kreutzberg G (1968). Displacement of synaptic terminals from regenerating motoneurons by microglial cells. Z Zellforsch Mikrosk Anat.

[35] Gehrmann J, Gold R, Linington C, Lannes-Vieira J, Wekerle H, Kreutzberg GW (1993). Microglial involvement in experimental autoimmune inflammation of the central and peripheral nervous system. Glia.

[36] Graeber MB, Kreutzberg GW (1986). Astrocytes increase in glial fibrillary acidic protein during retrograde changes of facial motor neurons. J Neurocytol.

[37] Kreutzberg GW (1996). Microglia: a sensor for pathological events in the CNS. Trends Neurosci.

[38] Raivich G, Bohatschek M, Kloss CU, Werner A, Jones LL, Kreutzberg GW (1999). Neuroglial activation repertoire in the injured brain: graded response, molecular mechanisms and cues to physiological function. Brain Res Brain Res Rev.

[39] Bergerot A, Shortland PJ, Anand P, Hunt SP, Carlstedt T (2004). Co-treatment with riluzole and GDNF is necessary for functional recovery after ventral root avulsion injury. Exp Neurol.

[40] Eggers R, Hendriks WT, Tannemaat MR, van Heerikhuize JJ, Pool CW, Carlstedt TP, Zaldumbide A, Hoeben RC, Boer GJ, Verhaagen J (2008). Neuroregenerative effects of lentiviral vector-mediated GDNF expression in reimplanted ventral roots. Mol Cell Neurosci.

[41] Rodrigues Hell RC, Silva Costa MM, Goes AM, Oliveira AL (2009). Local injection of BDNF producing mesenchymal stem cells increases neuronal survival and synaptic stability following ventral root avulsion. Neurobiol Dis.

[42] Kalinina NI, Sysoeva VY, Rubina KA, Parfenova YV, Tkachuk VA (2011). Mesenchymal stem cells in tissue growth and repair. Acta Nat.

[43] Lopatina T, Kalinina N, Karagyaur M, Stambolsky D, Rubina K, Revischin A, Pavlova G, Parfyonova Y, Tkachuk V (2011). Adipose-derived stem cells stimulate regeneration of peripheral nerves: BDNF secreted by these cells promotes nerve healing and axon growth de novo. PLoS One.

[44] Rubina K, Kalinina N, Efimenko A, Lopatina T, Melikhova V, Tsokolaeva Z, Sysoeva V, Tkachuk V, Parfyonova Y (2009). Adipose stromal cells stimulate angiogenesis via promoting progenitor cell differentiation, secretion of angiogenic factors, and enhancing vessel maturation. Tissue Eng Part A.

[45] Petter-Puchner AH, Froetscher W, Krametter-Froetscher R, Lorinson D, Redl H, van Griensven M (2007). The long-term neurocompatibility of human fibrin sealant and equine collagen as biomatrices in experimental spinal cord injury. Exp Toxicol Pathol.

