# Supplementary material for: Neuroprotection and immunomodulation following intraspinal axotomy of motoneurons by treatment with adult mesenchymal stem cells
Source: J Neuroinflammation. 2018 Aug 14;15:230. doi: 10.1186/s12974-018-1268-4 (PMC6092804; doi:10.1186/s12974-018-1268-4)
Supplement: Supplementary file 1 — Table S1. Primary antibodies used for Flow Cytometry. (DOCX 13 kb) [file 12974_2018_1268_MOESM1_ESM.docx]

**Table S1.** Primary antibodies used for Flow Citometry.

| **Antibody** | **Supplier** | **Host Animal** | **Product Code** | **Concentration** |
| --- | --- | --- | --- | --- |
| CD90 | BD Biosciences | Mouse | 554892 | 1:250 |
| CD54 | BD Biosciences | Mouse | 554967 | 1:250 |
| CD73 | BD Biosciences | Mouse | 551123 | 1:250 |
| RT1A (MHCI) | BD Biosciences | Mouse | 554917 | 1:250 |
| CD45 | BD Biosciences | Mouse | 555480 | 1:250 |
| CD11b/c | BD Biosciences | Mouse | 554859 | 1:250 |
| CD34 | Santa Cruz | Mouse | SC7324 | 1:200 |
