# Supplementary material for: Neuroprotection and immunomodulation following intraspinal axotomy of motoneurons by treatment with adult mesenchymal stem cells
Source: J Neuroinflammation. 2018 Aug 14;15:230. doi: 10.1186/s12974-018-1268-4 (PMC6092804; doi:10.1186/s12974-018-1268-4)
Supplement: Supplementary file 2 — Table S2. Primary antibodies used for Immunohistochemistry. (DOCX 13 kb) [file 12974_2018_1268_MOESM2_ESM.docx]

**Table S2.** Primary antibodies used for Immunohistochemistry.

| **Antibody** | **Supplier** | **Host Animal** | **Product Code** | **Concentration** |
| --- | --- | --- | --- | --- |
| GFAP | Abcam | rabbit | AB7779 | 1 : 1500 |
| Synaptophysin | Millipore | mouse | MAB5258 | 1 : 1500 |
| GAP-43 | Millipore | rabbit | AB5220 | 1 : 500 |
| Iba-1 | Wako | rabbit | 019-19741 | 1 : 700 |
| Arginase-1 | Santa Cruz | rabbit | SC-20150 | 1 : 700 |
| BDNF | Millipore | rabbit | AB1779 | 1 : 500 |
