# Supplementary material for: Neuroprotection and immunomodulation following intraspinal axotomy of motoneurons by treatment with adult mesenchymal stem cells
Source: J Neuroinflammation. 2018 Aug 14;15:230. doi: 10.1186/s12974-018-1268-4 (PMC6092804; doi:10.1186/s12974-018-1268-4)
Supplement: Supplementary file 3 — Table S3. Assays used for qPCR. (DOCX 13 kb) [file 12974_2018_1268_MOESM3_ESM.docx]

**Table S3.** Assays used for qPCR.

| **Gene** | **Thermofisher Product Code** |
| --- | --- |
| GAPDH | Rn01775763_g1 |
| HPRT1 | Rn01527840_m1 |
| VEGF | Rn01511601_m1 |
| BDNF | Rn02531967_s1 |
| Arg-1 | Rn00691090_m1 |
| Inos2 | Rn00561646_m1 |
| TNF-α | Rn01525859_g1 |
| IL-6 | Rn01410330_m1 |
| IL-1β | Rn00580432_m1 |
| IL-10 | Rn00563409_m1 |
| IL-4 | Rn01456866_m1 |
| IL-13 | Rn00587615_m1 |
| TGF-β | Rn00572010_m1 |
