# Supplementary figures and images for: Neuroprotection and immunomodulation following intraspinal axotomy of motoneurons by treatment with adult mesenchymal stem cells
Source: J Neuroinflammation. 2018 Aug 14;15:230. doi: 10.1186/s12974-018-1268-4 (PMC6092804; doi:10.1186/s12974-018-1268-4)

**A.** CD90

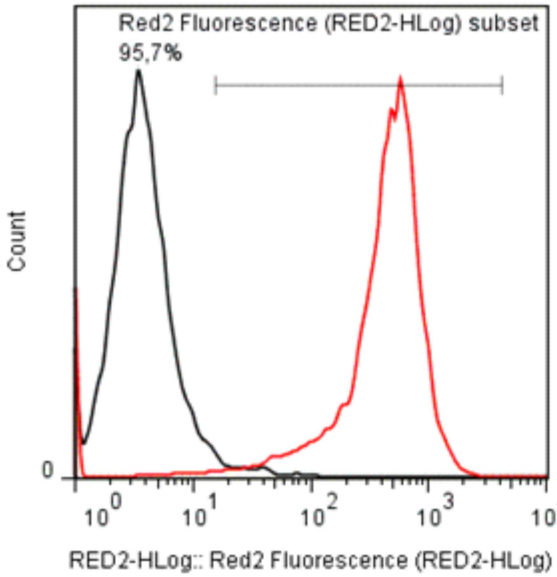

**B.** CD54

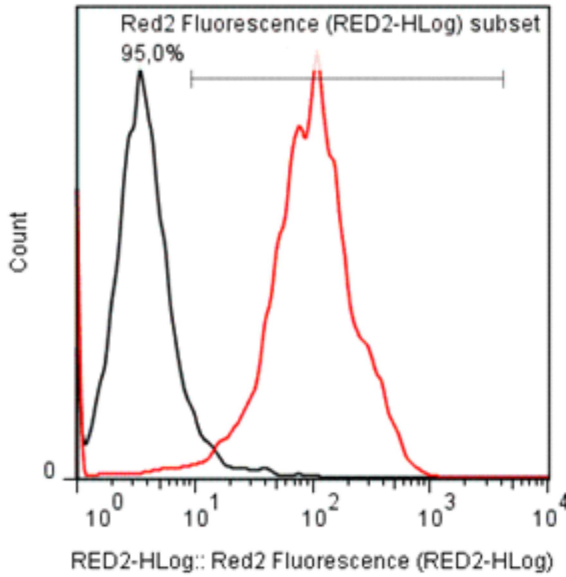

**C.** CD73

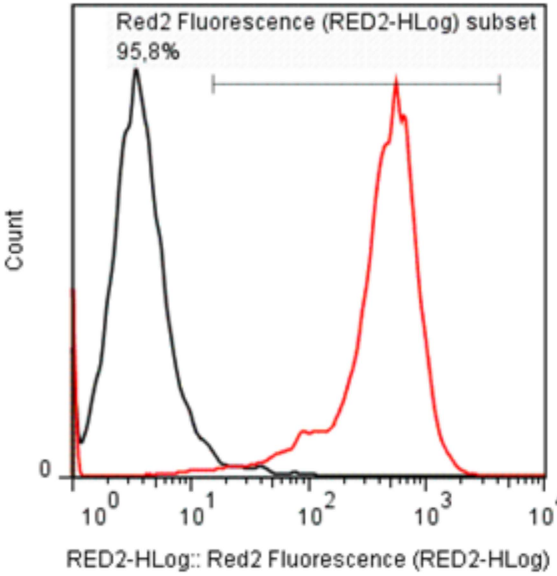

**D.** RT1A

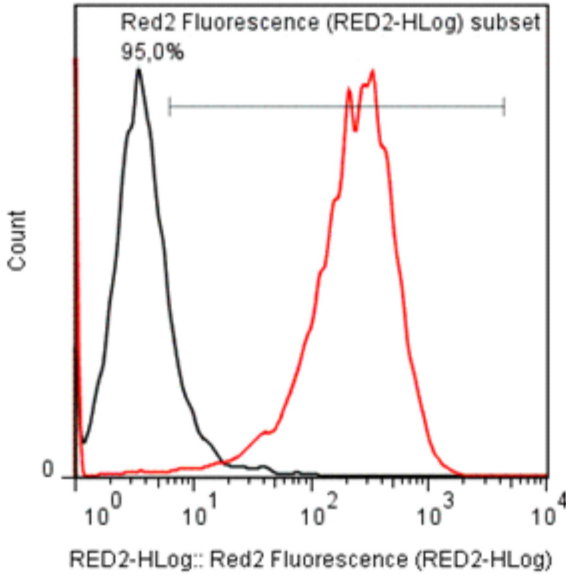

**E.** CD45

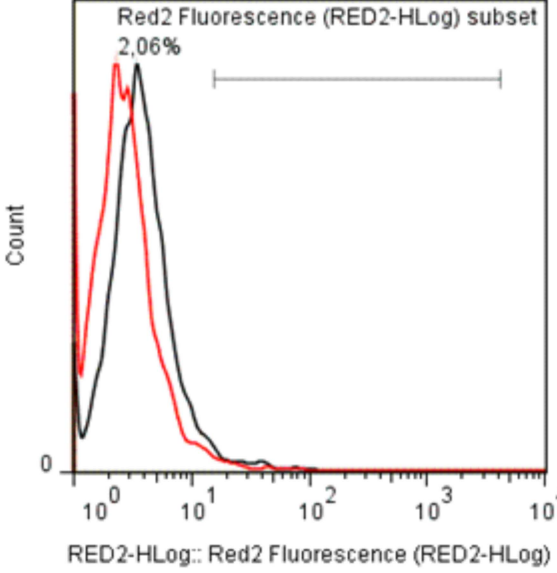

**F.** CD11b/c

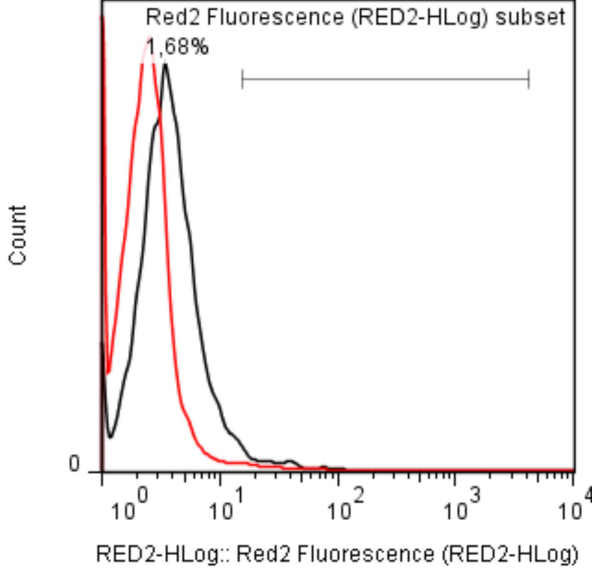

**G.** CD34

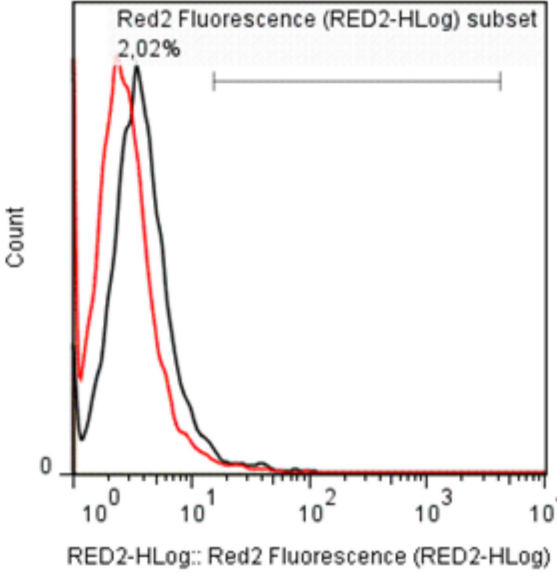

**H.**

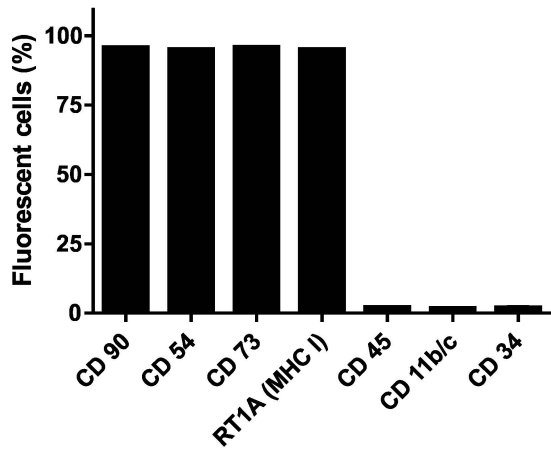

Supplement: Supplementary file 4 — Figure S1. Phenotypic analysis of the MSCs by flow cytometry. (A-G) Histograms of the mean fluorescence intensity (GRN-HLog: green fluorescence) versus the number of events (counts). (A) CD90. (B) CD54. (C) CD73. (D) MHCl (RT1A). (E) CD45. (F) CD11b/c. (G) CD34. (H) Graphic representation of the percentage of fluorescent cells labeled with each antibody. (PDF 826 kb) [file 12974_2018_1268_MOESM4_ESM.pdf]
